# Ageing opioid users’ increased risk of methadone-specific death in the UK

**DOI:** 10.1016/j.drugpo.2018.02.005

**Published:** 2018-05

**Authors:** Matthias Pierce, Tim Millar, J. Roy Robertson, Sheila M. Bird

**Affiliations:** a Centre for Biostatistics, School of Health Sciences, University of Manchester, Manchester M13 9PL, United Kingdom; b Centre for Mental Health and Safety, School of Health Sciences, University of Manchester, Manchester M13 9PL, United Kingdom; c Usher Institute of Population Health Sciences and Informatics, University of Edinburgh, Edinburgh EH16 4UX, United Kingdom; d MRC Biostatistics Unit, University of Cambridge, School of Clinical Medicine, Institute for Public Health, Cambridge CB2 0SR, United Kingdom; e Department of Mathematics and Statistics, University of Strathclyde, Glasgow G1 1XH, United Kingdom

**Keywords:** Drug-related deaths, Methadone-specific deaths, Age-relatedness, Gender, Risk behaviours, Pooling, Age-related hazard ratios

## Abstract

**Background:**

The first evidence that the hazard ratio (HR) for methadone-specific death rises more steeply with age-group than for all drug-related deaths (DRDs) came from Scotland’s cohort of 33,000 methadone-prescription clients. We aim to examine, for England, whether illicit opioid users’ risk of methadone-specific death increases with age; and to pool age-related HRs for methadone-specific deaths with those for Scotland’s methadone-prescription clients.

**Methods:**

The setting is all services in England that provide publicly-funded, structured treatment for illicit opioid users, the methodology linkage of the English National Drug Treatment Monitoring System and mortality database, and key measurements are DRDs, methadone-specific DRDs, or heroin-specific DRDs, by age-group and gender, with proportional hazards adjustment for substances used, injecting status and periods in/out of treatment.

**Results:**

Linkage was achieved for 129,979 adults receiving prescribing treatment modalities for opioid dependence during April 2005 to March 2009 and followed-up for 378,009 person-years (pys).

There were 1,266 DRDs: 271 methadone-specific (7 per 10,000 pys: irrespective of gender) and 473 heroin-specific (15 per 10,000 pys for males, 7 for females). Methadone-specific DRD-rate per 10,000 person-years was 3.5 (95% CI: 2.7–4.4) at 18–34 years, 8.9 (CI: 7.3–10.5) at 35–44 years and 18 (CI: 13.8–21.2) at 45+ years; heroin-specific DRD-rate was unchanged with age.

Relative to 25–34 years, pooled HRs for UK clients’ methadone-specific deaths were: 0.87 at <25 years (95% CI: 0.56–1.35); 2.14 at 35–44 years (95% CI: 1.76–2.60); 3.75 at 45+ years (95% CI: 2.99–4.70).

**Conclusion:**

International testing and explanation are needed of UK’s sharp age-related increase in the risk of methadone-specific death. Clients should be alerted that their risk of methadone-specific death increases as they age.

## What is known already

• Record-linkage studies internationally have shown the value of opioid substitution therapy as a treatment which reduces substantially clients’ risk of drug-related death (DRD); also that DRD-rates are lower for female opioid users and increase with age beyond 35 years but that the female advantage is much reduced for older clients.
• Despite harm reduction measures, such as opioid substitution therapy, UK’s DRDs have increased markedly in the past decade, in a strongly age-related manner.
• One powerful record-linkage study on Scotland’s methadone-prescription clients in 2009–2013 has shown that their adjusted hazard ratios for methadone-specific DRD increased sharply by age-group, irrespective of gender.

## What this study adds

• By analysing the opioid-specificity of deaths for England’s National Drug Treatment Monitoring System (NDTMS) cohort of nearly 130,000 opioid users who started a prescribing treatment modality, predominantly methadone, during 1 April 2005 to 31 March 2009, we confirmed that their hazard ratios for methadone-specific DRDs also increased sharply by age-group.
• Importantly, nearly half of the cohort’s person-years were aged 35+ years; and age-effects persisted after adjustment for risk-behaviours.
• By pooling results from the two major UK studies, we showed that opioid-dependent clients’ hazard ratio for methadone-specific death nearly doubled at 35–44 years (compared with 25–34 years); and quadrupled at 45+ years.

## Introduction

In the past decade, record-linkage studies in the UK and internationally have not only shown the value of opioid substitution therapy as a treatment which reduces substantially clients’ risk of drug-related death (DRD), by half (95% CI: 0.38 to 0.51) for those with a history of injecting (Pierce et al., 2016); but also that DRD-rates are lower for female opioid users; increase with age beyond 35 years; and yet the female advantage is reduced for older clients (Bird, Robertson, & Strang, 2010; Cornish, Macleod, Strang, Vickerman, & Hickman, 2010; Cousins et al. 2011; Degenhardt et al., 2011; Kimber et al., 2010; King, Bird, Overstall, Hay, & Hutchinson, 2013; King, Bird, Overstall, Hay, & Hutchinson, 2014; Merrall et al. 2012; Pierce et al., 2016; Pierce, Bird, Hickman, & Millar, 2015; Strang, Hall, Hickman, & Bird, 2010).

To assess the role of prescribed methadone in explaining the above demographic influences, Gao et al. (2016) considered age-group and gender, in addition to prescription source (general practitioner, other-source) and quintile for the quantity of prescribed methadone, as being potentially informative about the 361 methadone-specific DRDs experienced by 33,000 methadone-prescription clients in Scotland during 121,000 person-years of follow-up in 2009 to 2013. Their analysis revealed a steeply increased hazard by age-group, irrespective of gender (which was not prognostic) and that the top quintile for the baseline quantity of prescribed methadone conferred additional hazard for methadone-specific DRDs.

Relative to 25–34 year olds in the Scottish methadone-prescription cohort, the adjusted hazard ratio (HR) for methadone-specific deaths was 0.5 (95%CI: 0.3–1.0) for those aged under 25 years, 1.9 (95% CI: 1.5–2.4) at 35–44 years and 2.9 (95% CI: 2.2–3.9) at 45+ years of age. Eleven percent of Scotland’s methadone-prescription clients were aged 45+ years.

The first to demonstrate how steeply the risk of methadone-specific death increases with client-age, Scotland’s methadone-prescription cohort had the advantage of a national protocol in Scotland for toxicological reporting at forensic autopsy so that the specified opioids were implicated in, not merely present at, DRD. However, substantial numbers of Scotland’s two million methadone prescriptions issued over four years lacked a Community-Health Index (CHI)-number, meaning that they were not readily linkable to individual clients. For that reason, Scottish clients’ periods on/off prescribed methadone could not be analysed (Gao et al., 2016). Nonetheless, an estimated 82% of Scotland’s methadone clients were linkable to mortality-records because they had at least one CHI-identified prescription (Gao et al., 2016).

Besides calling for a better understanding of methadone’s pharmacodynamics in older clients, many with potential confounders such as progressive physical or mental ill-health, Gao et al. (2016) noted that, since 2006, electrocardiograms have been recommended by UK’s Medicines and Healthcare products Regulatory Authority for older or persistent methadone clients on higher doses. This recommendation was made to detect prolongation of that part of the heart’s normal electrical cycle known as the QT interval because, unlike buprenorphine, methadone – alone and with other drugs commonly used for co-morbidities, especially mental health conditions: antidepressants, antipsychotics, antibiotics (macrolides, quinolones, azoles), antiarrhythmics, protease inhibitors and the loop diuretic furosemide (Romero et al., 2016) – is known to prolong the QT interval; and prolongation increases the risk of torsades de pointes and sudden cardiac death, yet leaves no detectable trace at autopsy. Other risk factors for corrected QT prolongation include age-related co-morbidities such as renal impairment, heart or liver disease; and being female, see Gao et al. (2016).

Before diving too deeply into confounders, as above, in explanation for the strongly age-related increase in Scottish clients’ hazard of methadone-specific death, we considered that it was important first to test the Scottish results. England’s National Drug Treatment Monitoring System (NDTMS) cohort of nearly 130,000 opioid users who had started a prescribing treatment modality during 1 April 2005 to 31 March 2009 enabled such testing. The calendar period for the NDTMS cohort was immediately preceding Scotland’s (Pierce et al. 2016), thereby also preceding the heroin drought of 2010; 47% of the NDTMS cohort’s person-years were aged 35+ years, similar to 48% at baseline for Scotland’s methadone-prescription cohort. Importantly, information on NDTMS clients’ periods in/out of treatment, declared injecting and misuse of alcohol, benzodiazepines and other drugs (Pierce et al. 2016) could be taken into account in addition to age-group and gender. The English cohort’s gender and age-specific mortality from causes other than DRDs has been reported elsewhere (Pierce et al., 2015).

In this paper, for NDTMS opioid-user clients who received at least one day of opioid agonist prescribing (OAP), we aimed to:

i) document the influence of demographic risk factors (age-group; gender) on OAP clients’ HR for DRD, methadone-specific DRD, heroin-specific DRD, having adjusted also for clients’ time-dependent declared injecting (ever) and past-month misuse of alcohol, benzodiazepines, and other drugs;
ii) repeat the above analysis with adjustment also for periods in/out of treatment;
iii) pool age-related HRs for methadone-specific deaths from the Scotland’s methadone-prescription cohort and England’s OAP cohort.

## Methods

### Data

**Fig. 1:**
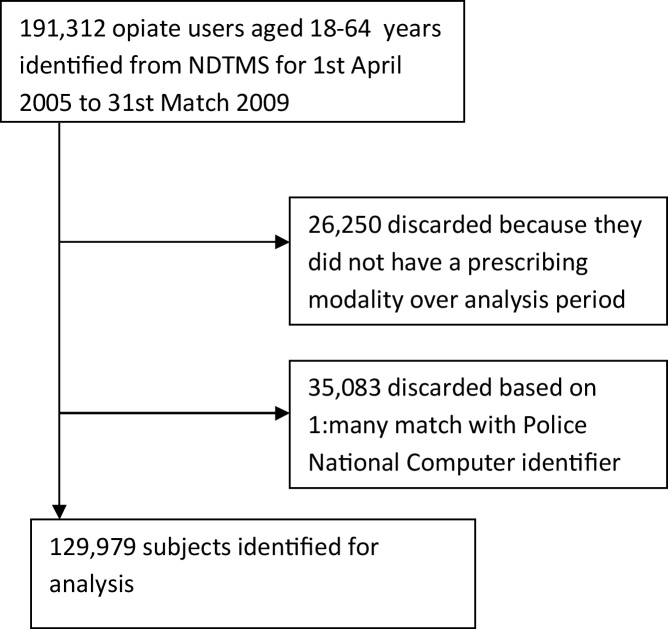
Flow diagram of case-selection for the analysis cohort.

The National Drug Treatment Monitoring System (NDTMS) provides details on all structured treatment for substance misuse provided in England. The cohort for this national record linkage study was identified from NDTMS records collected over the study period 1st April 2005 to 31st March 2009; with linkage to mortality records provided that the subject’s identifier was not in a many-to-one mapping, see Fig. 1. The Office for National Statistics (ONS) provided data on deaths occurring during the observation period which were registered by 30 September 2011. This allowed for delays in the registration process pending inquest verdicts.

Records in NDTMS are created for each treatment modality received by a given patient. Treatment modalities were categorised, as in Pierce et al. (2016) into: OAP; psychosocial treatment; and residential treatment. A treatment episode is defined as an unbroken or contiguous series of treatment modalities.

Data were supplied at the episode-level on: self-reported illicit drug injecting status (past month); and an optional self-report of up to three additional problematic psychoactive substances. In NDTMS, prescribed opioid agonist was not differentiated as to methadone or buprenorphine, but the vast majority was methadone (Cornish et al., 2010).

Patients were included in the OAP cohort if they received at least one day of prescribing treatment over the study period and were aged 18 to 64 at their inclusion-date. The cohort analysed here is a sub-cohort of that described by Pierce et al. (2016) who checked thoroughly on the proportionality of DRD hazards.

The NDTMS cohort was linked to ONS mortality records. For each death record, the underlying cause of death is coded according to the ICD-10 framework with DRDs defined by ICD-10 codes according to ONS’s definition (Office for National Statistics, 2017). Additionally, for each death, the ONS created flags to indicate whether ‘methadone’ and/or ‘heroin’ and/or ‘buprenorphine’ were mentioned on the death certificate.

In England, the opioid-specificity of DRDs is determined by the drugs which are mentioned on the death-certificate. As the majority of DRDs are registered following a coroner’s inquest, the text on the coroner’s death certificate is used by ONS to code all of the substances involved in the death. Analogous to Gao et al. (2016), we define methadone-specific DRDs as those in which methadone was mentioned but neither heroin nor buprenorphine; and heroin-specific DRDs as those in which heroin was mentioned but neither methadone nor buprenorphine. If opioid was mentioned on a DRD death certificate without further specification, which occurs for around 5% of England’s opioid-related DRDs (Office for National Statistics, 2017), the death was counted as DRD; but neither as methadone-specific DRD nor as heroin-specific DRD. Similarly, any DRD which mentioned buprenorphine was counted as DRD; but neither as methadone-specific DRD nor as heroin-specific DRD. However, buprenorphine is so infrequently mentioned in England and Wales as to be not separately tabulated by ONS even for DRD registrations in 2016.

Treatment and mortality data were linked using a minimal identifier in each database (initials, date of birth, gender) and government region of usual residence. Prior analysis indicated that 22% of minimal identifiers in the treatment population were shared by one or more individuals on the criminal records database which contributed to the cohort’s derivational linkages (Pierce et al., 2016). These minimal identifiers were removed from the analysis cohort because the subject’s drug treatment record could not be reliably linked to his/her mortality record. The resulting OAP cohort comprised in 129,979 linked individuals, see Fig. 1.

Data were rendered anonymous to the research team, via irreversible encryption of identifying information, prior to their release by source organizations.

### Statistical analysis

Descriptive life-table analysis (per 10,000 person-years of follow-up) was undertaken for the outcomes: any DRD; DRD that mentioned methadone on the death certificate but neither heroin nor buprenorphine (methadone-specific DRD); and DRD that mentioned heroin on the death certificate but neither methadone nor buprenorphine (heroin-specific DRD).

Subjects entered the risk-set at their earliest OAP-treatment date during the period 1st April 2005 to 31st March 2009. They left the risk set at the earlier of their date of death, 65th birthday, or the end of data collection (31st March 2009).

Time-dependent covariates for the OAP cohort were time-updated age (categorised as 18–24; 25–34, 35–44, 45–64 years) and patient-reported behavioural covariates, collected at the beginning of each treatment episode and defined from their date of first report, namely: injecting status; problematic use of alcohol, benzodiazepines, crack cocaine, cocaine powder and amphetamines (the latter two substances combined due to a low level of reporting). A time-dependent covariate for being in/out of treatment was also created to indicate whether the patient was enrolled in any structured treatment (prescribing or otherwise) using the start and end dates for treatment modality.

For each outcome, cause-specific Cox proportional hazard ratios were estimated by censoring other deaths and using covariates as above. Interaction between age and gender was anticipated for DRDs (Pierce et al., 2015; King et al., 2013; King et al., 2014).

When pooling the age-related HRs for methadone-specific deaths from the Scottish and English cohorts, age-group specific weights were derived from the information contributed per age-group by each cohort; alternatively, a common weighting was derived from the total information across all three age-groups (<25 years; 35–44 years; 45+ years). Please see Supplementary Information for details of both calculations. In statistical terms, notice that the information which a cohort contributes about ln HR for a particular age-group (relative to 25–34 year olds) is defined by the reciprocal of the variance of the cohort’s estimated ln HR for that age-group.

## Results

England’s OAP cohort of 129,979 prescribing modality clients was followed-up for 378,009 person-years (pys) and experienced 1,266 DRDs, an overall rate of 33 DRDs per 10,000 pys (95% CI: 31.6–35.3), of which 271 were methadone-specific DRDs and 473 were heroin-specific.

Overall, the OAP cohort’s methadone-specific DRD-rate was 7 per 10,000 pys, irrespective of gender. By age-group, however, the methadone-specific DRD-rate was 3.5 (95% CI: 2.7–4.4) at 18–34 years, 8.9 (CI: 7.3–10.5) at 35–44 years and 18 (CI: 13.8–21.2) at 45+ years; whereas the heroin-specific DRD-rate of 12.5 per 10,000 pys was unchanged by age, but was significantly higher for males than females, see Table 1.

**Table 1:** Proportional hazard analysis: DRD, methadone only and heroin only mortality associated with covariates, for opioid users who had a prescribing treatment modality over the period 1 st April 2005 to 31 st March 2009. (N = 129,979; person-years (pys) = 378,009).

			DRD				Methadone only				Heroin only		
	Pys (k)	Deaths	CMR per 10k pys	Adjusted Hazard Ratio [95% CI]*	P value	Deaths	CMR per 10k pys	Adjusted Hazard Ratio [95% CI]*	P value	Deaths	CMR per 10k pys	Adjusted Hazard Ratio [95% CI]*	P value
Age													
18–24	33,588	74	22.0	0.92 [0.72, 1.18]	<0.001	14	4.2	1.26 [0.70, 2.27]	<0.001	36	10.7	0.99 [0.69, 1.41]	0.796
25–34	167,045	429	25.7	REF		57	3.4	REF		202	12.1	REF	
35–44	128,936	514	39.9	1.54 [1.35, 1.75]		115	8.9	2.60 [1.89, 3.57]		172	13.3	1.09 [0.89, 1.33]	
45+	48,530	249	51.3	2.08 [1.78, 2.44]		85	17.5	5.14 [3.66, 7.21]		63	13.0	1.11 [0.84, 1.48]	
Gender													
Male	257,948	971	37.6	REF	<0.001	188	7.3	REF	0.500	390	15.1	REF	<0.001
Female	120,151	295	24.6	0.73 [0.64, 0.83]		83	6.9	1.09 [0.84, 1.42]		83	6.9	0.49 [0.38, 0.62]	
Injecting, ever declared													
Yes	147,341	704	47.8	2.12 [1.88, 2.40]	<0.001	130	8.8	1.64 [1.27, 2.13]	0.001	277	18.8	2.47 [2.00, 3.04]	<0.001
No	194,901	418	21.5	REF		111	5.7	REF		135	6.9	REF	
Undeclared	35,857	144	40.2	1.95 [1.61, 2.36]		30	8.4	1.47 [0.98, 2.21]		61	17.0	2.51 [1.85, 3.40]	
Declared alcohol misuse													
Yes	45,557	258	56.6	1.68 [1.46, 1.93]	<0.001	48	10.5	1.44 [1.05, 1.98]	0.024	92	20.1	1.63 [1.29, 2.05]	<0.001
No	332,542	1008	30.3	REF		223	6.7	REF		381	11.5	REF	
Declared benzodiazepine misuse													
Yes	54,051	258	47.7	1.39 [1.21, 1.60]	<0.001	54	10.0	1.44 [1.07, 1.95]	0.017	95	17.6	1.34 [1.07, 1.69]	0.011
No	324,049	1008	31.1	REF		217	6.7	REF		378	11.7	REF	
Declared crack cocaine misuse													
Yes	136,746	469	34.3	0.97 [0.87, 1.10]	0.671	76	5.6	0.69 [0.52, 0.90]	0.006	170	12.4	0.91 [0.75, 1.10]	0.334
No	241,353	797	33.0	REF		195	8.1	REF		303	12.5	REF	
Declared cocaine/amphetamine misuse													
Yes	49,451	188	38.0	1.04 [0.89, 1.22]	0.582	41	8.3	1.15 [0.82, 1.61]	0.420	84	17.0	1.29 [1.01, 1.64]	0.038
No	328,649	1078	32.8	REF		230	7.0	REF		389	11.8	REF	

* Evidence for gender and age interaction − DRD model: p = .013; methadone only model: p = .350; heroin only model: p = .062.

None of the behavioural risk-factors in Table 1 had a materially different influence on heroin-specific versus methadone-specific DRDs, apart from having ever injected (a more accentuated HR for heroin-specific deaths). Table 2 shows that the doubling of DRD-risk when out-of-treatment partitions as a nearly quadrupled risk of heroin-specific DRD but non-significant 20% out-of-treatment reduction in methadone-specific DRD risk. Other covariate influences are unchanged from Table 1.

**Table 2:** Proportional hazard analysis: DRD, methadone only and heroin only mortality associated with treatment and covariates, for opioid users who had a prescribing treatment modality over the period 1 st April 2005 to 31 st March 2009. (N = 129,979; person-years (pys) = 378,009).

			DRD				Methadone only				Heroin only		
	Pys (k)	Deaths	CMR per 10k pys	Adjusted Hazard Ratio [95% CI]*	P value	Deaths	CMR per 10k pys	Adjusted Hazard Ratio [95% CI]*	P value	Deaths	CMR per 10k pys	Adjusted Hazard Ratio [95% CI]*	P value
Treat													
In	283,801	790	27.8	REF	<0.001	217	7.6	REF	0.122	226	8.0	REF	<0.001
Out	94,298	476	50.5	1.98 [1.76, 2.23]		54	5.7	0.79 [0.58, 1.07]		247	26.2	3.72 [3.09, 4.49]	
Age													
18–24	33,588	74	22.0	0.90 [0.70, 1.15]	<0.001	14	4.2	1.27 [0.71, 2.28]	<0.001	36	10.7	0.93 [0.65, 1.33]	0.379
25–34	167,045	429	25.7	REF		57	3.4	REF		202	12.1	REF	
35–44	128,936	514	39.9	1.57 [1.38, 1.78]		115	8.9	2.59 [1.88, 3.56]		172	13.3	1.13 [0.92, 1.39]	
45+	48,530	249	51.3	2.18 [1.86, 2.55]		85	17.5	5.08 [3.62, 7.13]		63	13.0	1.22 [0.92, 1.62]	
Gender													
Male	257,948	971	37.6	REF	<0.001	188	7.3	REF	0.531	390	15.1	REF	<0.001
Female	120,151	295	24.6	0.74 [0.65, 0.85]		83	6.9	1.09 [0.84, 1.41]		83	6.9	0.52 [0.41, 0.65]	
Injecting, ever declared													
Yes	147,341	704	47.8	2.17 [1.91, 2.45]	<0.001	130	8.8	1.63 [1.26, 2.12]	0.001	277	18.8	2.58 [2.09, 3.18]	<0.001
No	194,901	418	21.5	REF		111	5.7	REF		135	6.9	REF	
Undeclared	35,857	144	40.2	1.77 [1.46, 2.15]		30	8.4	1.51 [1.01, 2.28]		61	17.0	2.08 [1.54, 2.83]	
Declared alcohol misuse													
Yes	45,557	258	56.6	1.73 [1.51, 1.99]	<0.001	48	10.5	1.43 [1.04, 1.96]	0.026	92	20.1	1.75 [1.39, 2.21]	<0.001
No	332,542	1008	30.3	REF		223	6.7	REF		381	11.5	REF	
Declared benzodiazepine misuse													
Yes	54,051	258	47.7	1.43 [1.25, 1.65]	<0.001	54	10.0	1.43 [1.06, 1.94]	0.020	95	17.6	1.45 [1.15, 1.82]	0.002
No	324,049	1008	31.1	REF		217	6.7	REF		378	11.7	REF	
Declared crack cocaine misuse													
Yes	136,746	469	34.3	0.97 [0.86, 1.09]	0.631	76	5.6	0.69 [0.52, 0.90]	0.007	170	12.4	0.91 [0.75, 1.11]	0.345
No	241,353	797	33.0	REF		195	8.1	REF		303	12.5	REF	
Declared cocaine/amphetamine misuse													
Yes	49,451	188	38.0	1.06 [0.91, 1.24]	0.454	41	8.3	1.15 [0.82, 1.60]	0.429	84	17.0	1.33 [1.05, 1.69]	0.018
No	328,649	1078	32.8	REF		230	7.0	REF		389	11.8	REF	

The weights used for the Scottish methadone-prescription cohort in pooling age-group specific HRs (see Introduction for the Scottish results to be pooled) were 43% for under 25 years of age, 63% for 35–44 year olds, and 55% at 45+ years; alternatively, a common weighting of 58% may be preferred, which is based on the sum of information across age-groups. The alternatives agree inferentially, see Supplementary information.

Relative to clients aged 25–34 years, the pooled HRs for methadone-specific deaths across Scotland’s methadone-prescription client cohort and the English prescribing cohort from Table 1 were: under 25 years of age, 0.87 (95% CI: 0.56–1.35); at 35–44 years, 2.14 (95% CI: 1.76–2.60); and at 45+ years, 3.75 (95% CI: 2.99–4.70).

## Discussion

### Summary of main findings and recommendations

The OAP cohort allowed us to validate, for England, the novel record-linkage findings from Scotland that opioid using clients’ hazard of methadone-specific death rises steeply with age and is independent of gender. In addition, we have shown that these demographic influences hold up when adjustment is made for a triad of declared behavioural characteristics (injecting, misuse of alcohol and misuse of benzodiazepines) and for periods in/out of opioid substitution therapy, factors not addressed in Scotland’s methadone-prescription cohort.

Next, we pooled age-related HRs from the two national cohorts for added precision so that, relative to 25–34 year olds, the OAP clients’ pooled HRs for methadone-specific deaths in the UK were doubled at 35–44 years and nearly quadrupled at 45+ years.

We therefore recommend that consideration be given to reviewing older clients’ methadone dosage in the context of the patient’s cardiovascular, respiratory and liver or renal related co-morbidities (Gao et al., 2016); and prescribed medications, including for mental health problems (Independent Expert Group, 2017). Gao et al. (2016) recommended that a representative sample of 45+ year old methadone clients be offered electrocardiograms (ECG) to establish what proportion of them (1%, 5% or 10%) had prolongation of their corrected QT segment (by 60 milliseconds or to above 500 milliseconds). In the light of our evidence-synthesis, we propose that this surveillance should be extended to 35–44 year olds. We propose surveillance as the initial step because we recognise that methadone treatment centres currently may lack equipment to perform and qualified staff to interpret ECGs and yet may be clients’ only contact with health care systems.

Finally, we recommend that methadone clients can safeguard themselves and their older peers by carrying a naloxone-kit (Bird, McAuley, Perry, & Hunter, 2016; Parmar, Strang, Choo, Meade, & Bird, 2017) and ensuring that family and friends know how to intervene in the event of overdose.

### Comparability of cohorts from England and Scotland

The availability of a cohort with well over 100 methadone-specific DRDs is a considerable strength of England’s validation study. The OAP cohort of nearly 130,000 clients (1 April 2005–31 March 2009) and Scotland’s methadone-prescription cohort of 33,000 clients (1 July 2009–30 June 2013) were broadly similar in terms of age-distribution, number of methadone-specific deaths (271; 361) and average follow-up time. Scottish clients’ age-distribution was documented at baseline only (Gao et al., 2016); the English cohort’s was time-updated. Scotland’s DRD-classification, supported by a national protocol for toxicology at forensic autopsy, is based on drugs implicated in the death. England’s used the text on the coroner’s death certificate to code all of the substances involved in the death.

Scot;1;land’s methadone-prescription cohort had an overall DRD rate of 63 per 10,000 pys compared with the OAP cohort’s much lower rate of 33 DRDs per 10,000 pys. This difference may be partly accounted for by injection drug use being less prevalent in England (King et al., 2013; King et al., 2014). Under half the OAP clients had a history of injecting (declared or undeclared), a behavioural covariate which, as Table 1 showed, doubles clients’ hazard ratio for DRD. Unfortunately, information on past-history of injecting was not directly available for Scotland’s methadone-prescription cohort. Period effects on DRD-rates cannot be ruled out between 2005-2009 and 2009–2013, the second of which coincided with a re-focusing of the UK’s drug policy away from harm reduction towards accelerated recovery, an emphasis that has since been moderated in the UK Government’s 2017 Drugs Strategy (2017), see Her Majesty’s Government (2017). Importantly, the earlier period preceded the heroin drought of 2010.

### Limitations

For different reasons, the availability of unique identifiers limited linkage to about 80% of clients in the OAP cohort, see Fig. 1; and in Scotland’s methadone-client cohort (Gao et al., 2016). As detailed in Methods, we discarded 22% of identifiers from the OAP cohort where there was evidence that two or more individuals might share the same details. Nonetheless, it remains possible that some proportion of the remainder might also have shared details with other individuals in their region. Errors in matching within treatment records and in matching these to mortality records might potentially have occurred as a consequence of this but, insofar as these arose due to the random coincidence of the minimal identifying information that was available in the OAP cohort, these errors will have contributed noise to the analysis rather than exerting any systematic effect.

England, and many other countries, does not have a national protocol for toxicology and its reporting at forensic autopsy. Thus, England’s classification of methadone-specific deaths is on a less firm basis than Scotland’s.

Details of opioid agonist prescribing were absent for the English cohort: both which agonist (methadone versus buprenorphine) and the quantity prescribed. We do not know directly what proportion of OAP clients received methadone, although we expect the proportion to have been high, around 90% (Cornish et al., 2010). Scotland’s age-related HRs for methadone-specific DRD increased just as steeply whether prescribed quantity of methadone was, or was not, fitted. Hence, the OAP cohort’s absence of methadone dose does not undermine our validation. Importantly, the English OAP cohort’s additional adjustment for behavioural covariates and for periods in/out of treatment, see also Pierce et al. (2016), did not mitigate the steep age-relatedness in adjusted HRs for methadone-specific deaths either.

Unlike for demography, self-reporting of behavioural covariates could lead to HRs that are closer to 1 than would be the case without mis-classification and so some residual behavoural confounding cannot be ruled out.

The absence from both record-linkage studies of information on ageing clients’ co-morbidities and specific other prescriptions which may interact with methadone is a limitation to be addressed in further research. In making a case for the public benefit of linking-in methadone clients’ hospitalizations and specified other prescribing records, applicants can now cite, in their support, the UK’s evidence-synthesis on ageing opioid users’ risk of methadone-specific death.

### Further research

Similarly large national cohort studies of OAP clients, investigating how age-group and gender influence the opioid-specificity of client-deaths, are needed for robust generalizations outside of the UK.

Within the UK, a strong public interest case can now be made for more extensive record-linkage studies to include, besides mortality and DRDs’ opioid-specificity, OAP clients’ or methadone-prescription clients’ history of hospitalizations (by which to infer co-morbidities), diagnosed carriage of blood-borne viruses (particularly the most prevalent, Hepatitis C virus), and selected co-prescribing (including for mental health conditions).

Without research, as above, into possible confounders, we can only speculate, as did Gao et al. (2016), on the underlying cause for the now-confirmed steep age-gradient for methadone-specific DRDs: corrected QT prolongation, renal or liver impairment, or some other aspect of the pharmacology of methadone in older clients that makes for a longer half-life. Indeed, it is the report of one of the authors that some methadone patients have expressed to him the opinion that, as they age, they don’t seem to need as high a methadone dose (JRR, personal communication). In short, cardiac and pulmonary co-morbidities, as well as liver and renal disease, may contribute to methadone-specific DRDs.

As too little is known about the gender- and age-specific pharmacology of methadone in older opioid-dependent clients, we suggest a pharmacokinetic study that simultaneously assesses methadone’s pharmacokinetic properties in different settings of clients’ cardiac, respiratory, renal or liver impairment. Additionally, given the concern for sudden cardiac death linked to QTc prolongation and subsequent arrhythmias, extended monitoring in the form of Holter monitoring or loop recorders could further elucidate older clients’ risk when prescribed methadone.

## Conclusion

Only formal studies will resolve what can and should be done to moderate opioid dependent clients’ increased risk of methadone-specific death as they age. Consistent findings in two major UK cohorts represent sufficient grounds for methadone prescribers, at least in the UK, to alert clients that their risk of methadone-specific death increases as they age − for reasons that we do yet fully understand. Review of older clients’ methadone dose in the light of co-morbidities and prescribed other medications may be worthwhile with consideration given, for some clients, to switching their opioid substitution formulation (McCance-Katz, 2011; Jolley et al., 2015).

## Funding

Medical Research Council grant G1000021 for the NIQUAD (Nationally Integrated Quantitative Understanding of Drug-harms) Addictions Cluster. The funder had no role in the decision to publish this paper.

## Contributions

SMB proposed the validation study for England to MP & TM who are its guarantors; TM holds privacy access permission for linkage of National Drug Data Warehouse (2005–2009) to Office for National Statistics mortality records, which was conducted in autumn 2011 to allow 2.5 years for the delayed registration of drugs-related deaths. SMB & JRR had instigated inquiries into Scotland’s methadone prescribing and a possible link to Scotland’s increased number of methadone-specific deaths and been co-authors of the initial study which demonstrated increased hazard for older clients; MP re-programmed previous analyses of the English cohort to focus on methadone-specific deaths; all authors contributed to the writing, referencing and review of the manuscript.

## Data sharing statement

The authors are bound by the conditions imposed on their access to the Drugs Data Warehouse but can assist others in how to achieve similar access.

## Transparency

The lead author (as the manuscript’s guarantor) affirms that the manuscript is an honest, accurate, and transparent account of the study being reported; that no important aspects of the study have been omitted; and that any discrepancies from the study as planned (and, if relevant, registered) have been explained.

## Ethics/privacy access approval

Data were extracted from the Drug Data Warehouse for a cohort of opioid users, aged 18 to 64 years, actively using or being treated for opioid use, in England over the period 1 st April 2005 to 31 st March 2009. Deaths occurring in the cohort were established by case linkage to national mortality records. Data were rendered anonymous to the research team, via irreversible encryption of identifying information, prior to their release by source organisations.

Use of mortality records was approved by the Office for National Statistics Microdata Release Panel. Use of data from the Drug Data Warehouse was authorised by those organisations providing data. The NHS Central Office for Research Ethics Committees and The University of Manchester Research Ethics Committee advised that further approval was not required for a study of this type.

## Conflicts of interest

SMB served on Scotland’s National Naloxone Advisory Group and was one of three co-principal investigators for the MRC-funded N-ALIVE pilot Trial of naloxone-on-release. SMB holds GSK shares. JRR chaired Scotland’s National Forum on Drugs-related Deaths and serves on the committee which is updating UK’s opioid prescribing recommendation. SMB and JRR were co-authors of the Scottish study for which this study provides validation.
