# Supplementary material for: Ageing opioid users’ increased risk of methadone-specific death in the UK
Source: Int J Drug Policy. 2018 May;55:121–7. doi: 10.1016/j.drugpo.2018.02.005 (PMC6004035; doi:10.1016/j.drugpo.2018.02.005)
Supplement: Supplementary file 1 [file mmc1.docx]

**Supplementary Information on weights used for pooling the logarithm of hazard ratios (ln HRs) for methadone-specific deaths across Scotland’s methadone-prescription client cohort and England’s OAP cohort.**

**Under 25s**

*Scottish cohort HR:* 0.53 (95% CI: 0.27 – 1.04).

Standard error for ln HR is [ln 1.04 – ln 0.27]/3.92, or 0.344. Information on ln HR from the Scottish cohort is the reciprocal of its standard error * standard error, or 8.4496.

*English cohort HR:* 1.26 (95% CI: 0.70 – 2.27).

Standard error for ln HR is [ln 2.27 – ln 0.70]/3.92, or 0.300. Information on ln HR from the English cohort is the reciprocal of its standard error * standard error, or 11.1025.

Information sum is 8.4496 + 11.1025, or 19.5521. ***Relative weight of information in the Scottish cohort is 8.4496/19.5521, or 43%.***

**35-44 year olds**

*Scottish cohort HR:* 1.91 (95% CI: 1.50 – 2.44).

Standard error for ln HR is [ln 2.44 – ln 1.50]/3.92, or 0.1241. Information on ln HR is the reciprocal of its standard error * standard error, or 64.9154.

*English cohort HR:* 2.60 (95% CI: 1.89 – 3.57).

Standard error for ln HR is [ln 3.57 – ln 1.89]/3.92, or 0.1622 Information on ln HR is the reciprocal of its standard error * standard error, or 37.9903.

Information sum is 64.9154 + 37.9903, or 102.9057. ***Relative weight of information in the Scottish cohort is 64.9154/102.9057, or 63%.***

**45+ year olds**

*Scottish cohort:* 2.90 (95% CI: 2.14 – 3.93).

Standard error on the ln scale is [ln 3.93 – ln 2.14]/3.92, or 0.1551. Information is the reciprocal of standard error * standard error, or 41.5913.

*English cohort:* 5.14 (95% CI: 3.66 – 7.21).

Standard error on the ln scale is [ln 7.21 – ln 3.66]/3.92, or 0.1730. Information is the reciprocal of standard error * standard error, or 33.4276.

Information sum is 41.5913 + 33.4276, or 75.0189. ***Relative weight of information in the Scottish cohort is 41.5913/75.0189, or 55%.***

Applying the above derived weights, the pooled ln HRs per age-group and their associated standard errors are:

**< 25 years:** pooled ln HR is 0.43 * -0.635 + 0.57 * 0.231, or -0.141; standard error for pooled ln HR is sqrt (reciprocal of information sum, 1/19.5521) or 0.226. Hence, 95% CI for pooled ln HR is -0.584 to 0.302. ***Finally, HR is 0.87 (95% CI: 0.56 – 1.35).***

**35-44 years:** pooled ln HR is 0.63 * 0.647 + 0.37 * 0.956, or 0.761; standard error for pooled ln HR is sqrt (reciprocal of information sum, 1/102.9057) or 0.098. Hence, 95% CI for pooled ln HR is 0.568 to 0.954***. Finally, HR is 2.14 (95% CI: 1.76 – 2.60).***

**45+ years:** pooled ln HR is 0.55 * 1.0647 + 0.45 * 1.6371, or 1.322; standard error for pooled ln HR is sqrt (reciprocal of information sum, 1/75.0189) or 0.115. Hence, 95% CI for pooled ln HR is 1.096 to 1.548. ***Finally, HR is 3.75 (95% CI: 2.99 – 4.70).***

**Alternatively**

If a common weighting scheme across age-groups is preferred, then the ***running common weight*** ***for the Scottish cohort*** should be:

[***8.4496*** + ***64.9154*** + ***41.5913***] or ***114.9563*** divided by the sum of information across age-groups

[***19.5521*** + ***102.9057*** + ***75.0189***], which is **197.4767** and so the common weighting across age-groups for the Scottish cohort would be ***114.9563***/**197.4767** or ***58%*.**

Applying the common weight of 58% across age-groups, the pooled ln HRs per age-group and their associated standard errors are:

**< 25 years:** pooled ln HR is 0.58 * -0.635 + 0.42 * 0.231, or -0.271; standard error for pooled ln HR is sqrt (0.58 * 0.58 * 0.344 * 0.344 + 0.42 * 0.42 * 0.300 * 0.300) or 0.236. Hence, the 95% CI for pooled ln HR is -0.733 to 0.1915. *Finally, HR is 0.76 (95% CI: 0.48 – 1.21).*

**35-44 years:** pooled ln HR is 0.58 * 0.647 + 0.42 * 0.956, or 0.777; standard error for pooled ln HR is sqrt (0.58 * 0.58 * 0.1242 * 0.1241 + 0.42 * 0.42 * 0.1622 * 0.1622) or 0.099. Hence, 95% CI for pooled ln HR is 0.583 to 0.971. *Finally, HR is 2.17 (95% CI: 1.79 – 2.64).*

**45+ years:** pooled ln HR is 0.58 * 1.065 + 0.42 * 1.637, or 1.305; standard error for pooled ln HR is sqrt (0.58 * 0.58 * 0.1551 * 0.1551 + 0.42 * 0.42 * 0.1730 * 0.1730) or 0.1156. Hence, 95% CI for pooled ln HR is 1.078 to 1.532. *Finally, HR is 3.69 (95% CI: 2.94 – 4.63).*
